# Heterogeneity in populations of enterohaemorrhagic *Escherichia coli* undergoing d-serine adaptation

**DOI:** 10.1007/s00294-020-01130-7

**Published:** 2020-11-21

**Authors:** Nicky O’Boyle, Andrew J. Roe

**Affiliations:** Institute of Infection, Immunity and Inflammation, University of Glasgow, Glasgow, G12 8TA UK

**Keywords:** Adaptive evolution, EHEC, Mutation, Pathogenesis, Metabolism

## Abstract

Phenotypic and genetic heterogeneities are conserved features of prokaryotic populations. During periods of stress, this programmed diversity increases the likelihood that variants within the population will survive the adverse conditions, allowing for proliferation. Phenotypic heterogeneity can have a mutational or indeed a non-mutational basis as observed in bet-hedging strategies adopted by antibiotic-tolerant persister cells. Genetic variants can arise by phase variation (slip-strand mispairing, promoter inversions etc.), nucleotide polymorphisms resulting from replication errors or larger rearrangements such as deletions and insertions. In the face of selective pressures, these alterations may be neutral, beneficial or deleterious.

We recently described the genetic basis of tolerance to a normally toxic metabolite, d-serine (d-ser) in enterohaemorrhagic *E. coli* (EHEC). Here we summarize our work in the context of population dynamics, provide further discussion on the distinction between these tolerance mechanisms and the importance of heterogeneity for maximising adaptive potential.

Enterohaemorrhagic *E. coli* displays significant growth arrest upon exposure to millimolar concentrations of d-ser and this is associated with activation of an unusual SOS-like response characterized by induction of RecA expression (Connolly and Roe 2016). Moreover, its primary colonization apparatus—the locus of enterocyte effacement (LEE)-encoded type 3 secretion system is transcriptionally repressed by D-ser (Connolly et al. 2015), further supporting the notion that EHEC has evolved to become particularly incompatible with exposure to this amino acid (Fig. 1a). In our recent work (O’Boyle et al. 2020), we examined the effect of repeated exposure of EHEC to physiological concentrations of d-ser that were able to inhibit growth, thereby promoting adaptive evolution and the development of tolerance to d-ser. Whole-genome sequencing (WGS) and transcriptomics were then used to reveal the genetic basis of tolerance.

**Fig. 1:**
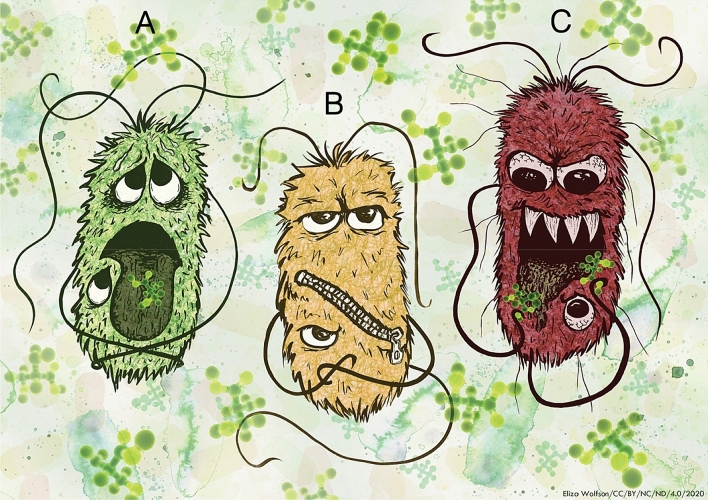
EHEC overcomes d-ser induced growth arrest by zipping the envelope shut or eating the poison. Upon exposure to millimolar d-ser (green ball and stick molecules) concentrations, wild-type EHEC (green cell) suffer growth arrest and activation of stress response (**a**). Growth arrest can be overcome by genetic disruption or transcriptional knock-down in inner membrane d-ser transporters (yellow cell), leading to zipping the cell envelope shut (**b**). Alternatively, mutations leading to the activation of the previously silent d-ser deaminase DsdA (red cell) allow EHEC to eat d-ser (the poison) and surmount the inhibition of growth normally caused by d-ser (**c**)

Complete d-ser tolerance was obtained through two distinct adaptive strategies: either by “zipping the cell envelope shut (Fig. 1b)” (blocking cytoplasmic accumulation of D-ser via the disruption of inner membrane transporters) or “eating the poison (Fig. 1c)” (constitutive activation of a d-ser deaminase that was normally locked in a silent state) (Fig. 1). “Zipping the envelope shut” was achieved primarily through inactivating single nucleotide polymorphisms (SNPs) in the d-ser transporter CycA (Cosloy 1973), allowing for growth in the presence of d-ser. These mutations appeared after as little as two successive 24-h batch cultures with distinct SNPs being observed in replicate evolution experiments. Importantly, inactivation of CycA alone resulted in partial tolerance and was subject to a refinement, observed after eight days of successive batch culture when stable, constitutive knock down in transcription of a second d-ser transporter SstT (Ogawa et al. 1998), resulted in growth rates in d-ser indistinguishable from those observed in the absence of d-ser (i.e., gain of complete tolerance). A similar refinement of an evolutionary innovation was seen during the evolution of aerobic citrate utilization under nutrient limitation in *E. coli*. Primary activation of *citT* by a rearrangement that led to control by the aerobically active *rnk* promoter facilitated the Cit + phenotype, but secondary refinement arising from a hyperactivating mutation in the *dctA* dicarboxylate transporter gene was required for full citrate utilisation (Quandt et al. 2014, 2015).

The alternative strategy identified in our study was to “eat the poison”. Diarrheagenic *E. coli* very rarely carry an intact *dsdCXA* locus (Moritz and Welch 2006; Connolly et al. 2015). Indeed, to date, we have never obtained a “wild-type” *E. coli* O157:H7 isolate containing both a functional-type three secretion system and the complete *dsdCXA* locus. Due to lack of the transcriptional activator DsdC, the deaminase encoded by *dsdA* does not respond to d-ser and remains silent (Connolly et al. 2015). We observed distinct genetic deletions allowing for full tolerance to d-ser by constitutive activation of d-ser deaminase via alternative upstream promoters. Again, the timing of emergence of these mutants was interesting. One full fitness mutant was acquired after only 2 days of successive batch culture while another was isolated on the tenth and final day of the experiment. Mutants were isolated on d-ser-containing plates to allow for discrimination of the tolerance phenotype. We believe that the mutation giving rise to full tolerance at day two was likely to have emerged after plating, as this mutant was not detected at any subsequent timepoint, and further full-fitness mutants adopting either transport or catabolic routes were not isolated until the eighth day of the experiment. Parallel adaptive evolution experiments with multiple populations can reveal differing transcriptional landscapes with convergent growth outcomes (Fong et al. 2005). In our work, we observed that this is also possible within a single population with full-fitness adaptive mutants displaying the “zip the envelope shut” and the “eat the poison” transcriptional profile and phenotype being isolated simultaneously on the final day of sampling. This is indicative of the co-existence of multiple adaptive lineages, a feature which has previously been described during long-term adaptation (Good et al. 2017) and highlights the inherent flexibility of *E. coli* populations in surmounting stress.

It should be noted that the sampling carried out in our study was far from exhaustive and it is likely that many additional evolutionary trajectories towards complete tolerance exist. The selective pressure imparted during differential identification of tolerant clones by plating on medium containing d-ser created desirable bias towards full-tolerance isolates, facilitating the analysis of fewer clones by WGS; however, this bias also prevented the analysis of neutral mutations. One can imagine further mutations within the population requiring more complex refinements before gaining a full tolerance phenotype. Deep sequencing of the mixed population during adaptation would more precisely reveal the dynamics of emergence and fixation of such mutations over time. Similarly, a comprehensive picture of the paths towards fitness can only be achieved through experimentation with replicate populations (Conrad et al. 2011). Our study involved only a short replicate experiment over a 48-h period as a proof of principle that distinct mutations in similar genes are selected for in multiple populations. It is quite likely that further replicates would identify novel adaptive routes to d-ser tolerance. For example, mutations were not observed in another transporter (YhaO/DlsT) that we have previously shown to functionally transport d-ser (Connolly et al. 2016). While further replicates may implicate disruption of DlsT in conferring tolerance to d-ser, our previous work showed that exposure to d-ser is required for maximal expression of DlsT and it is possible that without cytoplasmic accumulation of d-ser via CycA, expression of DlsT does not reach sufficient levels to cause growth inhibition in EHEC. The strength of selection has been shown to influence the evolutionary trajectory associated with overcoming a given selective pressure (Sanz-García et al. 2020). It should be noted that the 1 mM concentration used in our study was selected on the basis of being an approximation of the physiological concentration found in the bladder (Anfora et al. 2007), an environment unfavourable for EHEC. It is possible that different alleles would be adaptive in varying concentrations, an important consideration when trying to obtain a comprehensive appreciation of adaptive flexibility.

Finally, it should be noted that the adaptive routes described in our study confer advantages specifically in the in vitro system in which they were observed. As yet, it is unclear whether d-ser can exert such selective pressures within the host. It is also unclear what the consequences of these mutations would be for fitness within the host niche. Dietary consumption of compounds capable of inhibiting bacterial growth has been associated with the evolution of strains with enhanced pathogenicity (Collins et al. 2018). As adaptation to d-ser facilitated not only increased growth but also unexpectedly, resistance to repression of the type 3 secretion system essential for colonization, d-ser could apply selective pressure to pathogenic *E. coli* with important consequences for the outcome of infections.
